# Genome sequence of *Providencia stuartii* prov-sta1, isolated from the wasp *Nasonia vitripennis*

**DOI:** 10.1128/MRA.00430-23

**Published:** 2023-10-30

**Authors:** Runbiao Wu, Zhengyu Zhu, Guan-Hong Wang

**Affiliations:** 1 State Key Laboratory of Integrated Management of Pest Insects and Rodents, Institute of Zoology, Chinese Academy of Sciences, Beijing, China; 2 College of Life Sciences, Hebei University, Baoding, China; Indiana University, Bloomington, Indiana, USA

**Keywords:** bacteria, pathogenicity

## Abstract

*Providencia stuartii* prov-sta1 is a prevalent Gram-negative bacterium and dominant in the wasp *Nasonia vitripennis*. In this study, we present the draft genome sequence of *P. stuartii* prov-sta1, and the genome size is 4,380,152 bp in 183 contigs with a G+C content of 41.34%.

## ANNOUNCEMENT

*Providencia stuartii* is a Gram-negative bacterium that can be isolated from oil, fermentation dreg, insects, and even humans (1 – 4). The organism has received increasing attention because of its pathogenicity and drug resistance (5, 6). *Providencia* bacteria is the prevalent bacterial symbionts of the wasp, whether it is pathogenic to wasp host remains unclear (7). Therefore, analyzing the genome may provide a better understanding of the important roles of this bacterium in the physiology, development, behavior, reproduction, and evolution of the wasp.

We isolated and identified *P. stuartii* prov-sta1 from *N. vitripennis*. One adult female *N. vitripennis* was put into a 1.5 mL centrifugal tube and then washed with 1 mL 70% ethanol solution for 5 min, 1 mL 10% bleach solution for 2 min, and 1 mL sterile water for 2 min. The insect was homogenized in sterile phosphate buffer solution and plated 1× aliquots of the homogenate onto Luria-Bertani (LB) medium. The colonies of *P. stuartii* prov-sta1 appear gray or white with a smooth surface. The taxonomic identity was confirmed by amplifying the 16S rRNA sequence using the universal bacterial primers 27F and 1,492R.

For genome sequencing, individual colony was gently dipped with a white tip and placed in culture tubes containing 3 mL of LB liquid medium, followed by gyratory shaking (250 rpm) at 37°C for 24 h. The genome DNA was extracted using the TIANamp Genomic DNA Kit (Tiangen, China). The extracted bacterial genomic DNA The library was used for library construction for sequencing using the kits VAHTSTM Universal DNA Library Pren Kit (Vazyme, China) and VAHTSTM DNA Clean Beads (Vazyme, China). The bacterial genome undergoes steps such as interruption, ligand end repair and poly A addition, splice ligation, PCR amplification, and purification. The concentration of purified PCR products was quantified using Qubit 3.0 Fluorometer (Life Technologies, USA) and sent to Biomarker Technologies (Beijing, China) for whole-genome sequencing on an Illumina NovaSeq 6000 platform with an insert size of 350 bp, 150 bp paired-end (PE150) and around 2 G sequencing size. Raw data from high-throughput sequencing contain 12,059,802 reads and 1,790,022,318 nucleotides using FastQC v0.11.5 (8). Trimming and quality filtering were performed using fastp v0.23.2 (with parameters -z 4 -q 20 -u 30 -n 10) (9). Clean data with 1,790,021,709 nucleotides were assembled using SPAdes v3.15.4 (with parameters -k 21,335,577 --careful) (10). The assembly statistics were calculated using QUAST v5.2.0 (with default parameters) (11), obtaining 183 contigs, N50 and L50 values of 83,642 bp and 14, respectively. The total length of genome sequence of *P. stuartii* prov-sta1 contains 4,380,152 nucleotides, and G+C content is 41.34%, with approximately 408 fold coverage. We further assembled the contigs into Scaffold, which were 171 Scaffold and 4,381,193 bp in length. Sequence lengths shorter than 1,000 bp were removed, and gene annotation was performed using NCBI Prokaryotic Genome Annotation Pipeline (12), and the genome sequence of *P. stuartii* prov-sta1 contains 4,093 protein-coding genes, 4 rRNA genes, and 53 tRNA genes. Although the NCBI record recommended that the genome of this sequenced species belongs to *Providencia thailandensis* based on the sequenced sequence, the 16S sequencing and phylogenetic tree results support that this bacterium belongs to *Providencia stuartii*. We report the genome sequence of *P. stuartii* prov-sta1 will help understand the diversity and function of the symbiotic bacteria of wasps.

## Data Availability

Data have been deposited in the National Center for Biotechnology Information, and the raw Illumina sequencing data can be accessed via BioProject accession number PRJNA954321, BioSample accession number SAMN34139003, or Sequence Read Archive (SRA) accession number SRR24150500. The 16S rRNA gene amplicon sequence and assembled genome sequence were also deposited in GenBank with accession numbers OQ807159 and JASDXX000000000, respectively.
